# Divergent Biochemical Fractionation, Not Convergent Temperature, Explains Cellulose Oxygen Isotope Enrichment across Latitudes

**DOI:** 10.1371/journal.pone.0028040

**Published:** 2011-11-21

**Authors:** Leonel Sternberg, Patricia Fernandes Vendramini Ellsworth

**Affiliations:** Department of Biology, University of Miami, Coral Gables, Florida, United States of America; Lakehead University, Canada

## Abstract

Recent findings based on the oxygen isotope ratios of tree trunk cellulose indicate that the temperature of biomass production in biomes ranging from boreal to subtropical forests converge to an average leaf temperature of 21.4°C. The above conclusion has been drawn under the assumption that biochemically related isotopic fractionations during cellulose synthesis are not affected by temperature. Here we test the above assumption by heterotrophically generating cellulose at different temperatures and measuring the proportion of carbohydrate oxygen that exchange with water during cellulose synthesis and the average biochemical fractionation associated with this exchange. We observed no variation in the proportion of oxygen that exchange with different temperatures, which averaged 0.42 as it has been observed in other studies. On the other hand, the biochemical oxygen isotope fractionation during cellulose synthesis is affected by temperature and can be described by a 2^nd^ order polynomial equation. The biochemical fractionation changes little between temperatures of 20 and 30°C averaging 26‰ but increases at lower temperatures to values of 31‰. This temperature sensitive biochemical fractionation explains the pattern of cellulose oxygen isotope ratios of aquatic plants encompassing several latitudes. The observed temperature sensitive biochemical fractionation also indicates that divergent biochemical fractionation and not convergent leaf temperature explains the increase in oxygen isotope enrichment of cellulose across several biomes.

## Introduction

Oxygen isotope ratios of cellulose provide a powerful signal to decipher paleoclimate and ecohydrological processes of extant ecosystems. This tool becomes even more powerful when applied to the chronosequence of tree rings, making it possible to accurately date changes in climate or hydrology in fossil or present day samples. Recently, and based on the oxygen isotope ratios of stem cellulose from trees spanning several latitudes, it has been proposed that there is a convergence of optimal photosynthetic temperature during tree trunk biomass production [1]. Helliker and Richter [1] showed that cellulose oxygen isotope enrichment relative to ambient water increases with a decrease in mean annual temperature (or at higher latitudes). Helliker and Richter [1] ascribed this increase in cellulose oxygen isotope enrichment to a greater leaf water enrichment at geographical locations with lower mean annual temperatures (M.A.T.) compared to semitropical and temperate regions. They further argued that the leaf-air water vapor pressure gradient would have to be greater at higher latitudes to maintain the hypothetical pattern of leaf water enrichment. To sustain this high vapor gradient at higher latitudes, leaf temperature would have to be disproportionally greater relative to ambient temperature at higher latitudes. After modeling the leaf water isotopic enrichment necessary to explain the cellulose oxygen isotope ratios observations, they calculated a remarkably constant average leaf temperature of 21.4°C throughout all the latitudes. One of the assumptions critical to their conclusion was that the biochemical fractionations associated with the imprinting of the water oxygen isotope signal in cellulose is constant regardless of temperature.

The oxygen isotope enrichment of cellulose from stems and tree trunks relative to ambient water can be described by the following equation [2]:
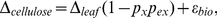
(1)in which Δ*_cellulose_* and Δ*_leaf_* are the respective oxygen isotope enrichment of cellulose and leaf water relative to the source water. The proportional amount of stem water relative to both leaf and stem water in the tissue where cellulose is synthesized is denoted as *p_x_*. Because the cellulose is usually extracted from stem or tree trunks with little possibility of having leaf water, we consider *p_x_* to be 1. As sucrose is translocated from the leaf to the stem for cellulose synthesis it exchanges approximately 40% of its oxygen with stem water before being converted to cellulose [3], [4], [5], [6]. This biochemical exchange between the oxygen in carbohydrates and water during cellulose synthesis is denoted as *p_ex_*. This exchange occurs primarily because carbonyl oxygen exchange with the oxygen in water during carbohydrate metabolism subject to an isotopic equilibrium effect [3], [7]. This exchange can be quite rapid for triose phosphates, but slower for pentoses and hexoses [8], [9]. One factor greatly contributing to this exchange is the futile cycle which occurs in the cytoplasm where glucose-6 phosphate can cycle quickly through fructose-1,6 Bisphosphate and triose phosphates [10]. Although *p_ex_* indicates the extent of exchange during cellulose synthesis, it does not specify the actual average isotopic fractionation for this process. The average biochemical fractionation factor for this exchange as well as other reactions leading to cellulose synthesis is specified be ε*_bio_*. The above equation (1) can be thought of as being composed of two major biological processes: physiological processes (Δ*_leaf_* and *p_x_*) and biochemical processes (*p_ex_* and ε*_bio_*) [11]. Helliker and Richter [1] assumed that one of the physiological processes (Δ*_leaf._*) in equation 1 increased with lower mean annual temperature in order to account for the increase in the oxygen isotopic enrichment of cellulose relative to source water. Although the authors added some variability in the biochemical factors (*p_ex_* and ε*_bio_*), they were considered constant regardless of latitude (temperature). Here we test whether the biochemical components of the above equation (*p_ex_* and ε*_bio_*) could be altered by temperature. For example, *p_ex_*, related to the above mentioned futile cycle, could decrease with lower temperatures. This would lower the carbonyl exchange reactions occurring at the tree trunk during cellulose synthesis, and promote a greater isotopically enriched leaf water signal in the cellulose synthesized in the tree trunk and a plausible explanation of the observed increase in Δ*_cellulose_* at lower temperatures. The other biochemical component, ε*_bio_*, involves an equilibrium reaction which could also be subject to temperature effects. Lower temperatures tend to increase the equilibrium fractionations non-linearly [12], which could also explain the observed pattern of oxygen isotope ratios in stem cellulose with latitude. We know of only two systematic studies of the temperature effects on the biochemical oxygen isotope fractionation during cellulose synthesis [13], [14]. The first one was a survey of aquatic marine plants ranging from Puerto Rico, the southernmost sample set, to the most northerly in Woods Hole, Massachusetts. In addition, the authors of this study grew plants in aquaria maintained at specified temperatures. Although there seemed to be a temperature effect on the wild collected plants, the aquaria experiment yielded mixed results. The other study is a dissertation [14] where 6 different plant species were grown at 20°C or 30°C daytime temperature and analyzed for oxygen isotope fractionation. The authors found no consistent pattern for each species. The 20 to 30°C temperature range is relatively small considering that mean annual temperatures in some locations where trees grow can be in the range of −5°C. We note that many of the plants included in the Helliker & Richter [1] study were growing in ambient temperatures below 20°C.

Here we test for temperature effects in the exchange rate (*p_ex_*) and biochemical fractionation (ε*_bio_*) in a previously used system which mimics synthesis of cellulose in a tree trunk: cellulose synthesis in germinating wheat seeds in complete darkness [15], [16]. During germination of wheat seeds, starch, the primary carbon storage of the seed, is broken down to glucose which is principally synthesized to sucrose [17]. The sucrose is then translocated to the site of cellulose synthesis and used as a substrate. Previous measurements show a good agreement between *p_ex_* measured with this system and those measured in wild and hydroponically grown trees [5], [6]. We germinated wheat seeds in the dark with waters showing different ^18^O enrichment at various temperatures. After growth we extracted cellulose and water from the culture flasks and observed the relationship between *p_ex_* and ε*_bio_* versus temperature. Specifically, we tested the hypothesis that *p_ex_* will decrease and ε*_bio_* will increase with lower temperatures. We then used these results to test whether a temperature dependent ε*_bio_* is consistent with those observed in aquatic plants growing at different temperatures [13], [18], [19] and if it might lead to a different conclusion than that of Helliker and Richter [1] regarding convergent leaf temperature.

## Materials and Methods

### Heterotrophic Cellulose Synthesis

We germinated wheat seeds and allowed the seedlings to develop from 9 to 30 days in the dark and in this way generate cellulose heterotrophically from the stored starch substrate. For a particular temperature seedlings were grown in 5 culture flasks each having water with different oxygen isotope ratios (∼0‰, 20‰, 40‰, 60‰ and 80‰). Seedlings were grown at 5°C, 10°C, 15°C, 20°C, 25°C and 30°C. After growth two aliquots of the agar medium were separated for water extraction and subsequent isotope analysis. Seedlings were separated from the remaining seeds, washed and dried at 50°C for several days. Dried seedlings were ground and cellulose extracted as in Leavitt & Danzer [20].

### Isotope Analysis

Water from the different cultures was analyzed for oxygen isotope ratios using the carbon dioxide equilibration method as in Vendramine & Sternberg [21]. Half a milliliter of water was sealed in small vials with a septum cover (LabCo, Buckinghamshire, England). Vials were placed in an automated analytical system (Multiflow, Elementar, Hanau, Germany) and flushed with a 5% CO_2_/He mixture for 3 minutes and allowed to equilibrate for 48 hours at room temperature. After equilibration an aliquot of the equilibrated CO_2_ was removed passed through a GC and introduced into the mass spectrometer (Isoprime, Cheadle, England) for isotopic analysis. Cellulose was analyzed by sealing ∼0.5 mg in silver capsules (Elementar Americas, New Jersey, U.S.A.) and introduced into a reaction vessel containing glassy carbon, topped off with 0.5 g of nickelized carbon (Elementar Americas, New Jersey, U.S.A.) and kept at 1080°C. Gasses from this reaction mainly nitrogen and carbon monoxide were separated in a GC and the CO introduced into the mass spectrometer to be analyzed for the oxygen 18 abundance. Oxygen isotope ratios are expressed as:
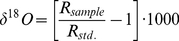
(2)Where R_sample_ and R_standard_ are the ^18^O/^16^O ratio of sample and SMOW reference respectively. The precision of analysis for water samples is typically ±0.1‰ and for the cellulose is ±0.3‰.

### Data Analysis

#### Calculation of p_ex_ and ε_bio_ from wheat germination experiments

We regressed the δ^18^O values of cellulose versus that of the media water for each temperature and compared the slopes (denoted here as *p_ex_*) of each relationship for different temperatures using an ANCOVA test. The comparison of slopes between temperatures was Bonferroni corrected for 15 comparisons.

Since we observed only one significant difference between the slopes of the above linear regressions and no significant trend with temperature, we used a value of *p_ex_* of 0.42, the average of all slopes observed here and in other literature (see Cernusak et al. [6] for a compilation of studies) to calculate ε*_bio_* with the following equation:
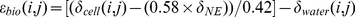
(3)Where ε*_bio_ (i,j)* is the average biochemical fractionation for the water isotopic enrichment level i and the temperature j and *δ_cell_ (i,j)* and *δ_water_ (i,j)* are the oxygen isotope ratios of cellulose and water for the seedling culture with water enrichment i and temperature j. If ^18^O is randomly distributed in the glucose moieties of the starch substrate, one could determine the value of the non-exchangeable oxygen (δ_NE_) by analyzing the starch substrate in the wheat seeds before germination. However, previous studies indicate that ^18^O is probably not randomly distributed in the glucose moieties of starch [16]. We, therefore chose a value of 33.4‰ for *δ_NE_*, which is similar to previous measurements [16] and gives the frequently observed value of ε*_bio_* of 27‰ at 25°C. If we erred in the value of δ_NE,_ our absolute values of fractionations are incorrect, but the observed trends with temperature are robust. We opted for the above method of calculating *ε_bio_*, rather than by the intercept method of the linear regression between *δ_cell_* versus *δ_water_* for a given temperature [3], as the intercept is highly biased towards cellulose grown in water with isotope ratios closer to 0. However, we show the results of the intercept method of calculation in Table S1. By the calculation method used here all cellulose cultures, regardless of water isotopic enrichment level, at a given temperature share equally towards the calculation of *ε_bio_*. We tested for temperature and water enrichment effects on ε_bio_ with a two way ANOVA to determine if there were significant differences between ε*_bio_* at different temperatures or/and at different culture water isotopic enrichment values. We derived an equation describing the relationship between average ε*_bio_* and temperature by a regression analysis of the average of ε*_bio_* for each temperature on temperature and calculating a best fit 2^nd^ order polynomial equation.

#### Calculation of Δ_cell_ for Aquatic plants

We selected three studies where oxygen isotope ratios of cellulose from aquatic plants were determined along with the lake/ocean water they grew in at several geographical locations [13], [18], [19]. Two of these studies [13], [18] also grew aquatic plants under different temperatures in aquaria, but aquarium data was only used from the Sauer et al. study [18], since only in this study it was certain that biomass was produced during the temperature treatment and no reserves from its previous growth were used to produce biomass. For the Sternberg study [19], lake water and the cellulose oxygen isotope ratios as well as the specific location and altitude of sampling were given. We derived temperature from the closest weather stations. In the case of Andean samples in the Sternberg [19] study, we used data from the closest station and corrected for altitude differences using the temperature lapse rate of intermediate to dry and saturated air. For the Sauer study [19] we assumed that plants were operating at a minimum temperature of 5°C. This is the optimal photosynthetic temperature observed in Antarctic lichen [22]. The DeNiro and Epstein survey [13] provided the growing season temperature for each site where marine plants were collected. Further information on these samples can be found in the supplementary material (Table S2). Since there is no transpiration in aquatic plants *Δ_cell_* = *ε_bio_*. We subtracted the δ^18^O values of lake/sea water/aquarium water from those of cellulose to derive the *Δ_cell_*, plotted *Δ_cell_* versus water temperature, and used regression analysis to calculate the best fit 2^nd^ order polynomial equation.

#### Re-evaluation of Tree Trunk Cellulose Oxygen Isotope data

We used data reported in the supplemental of Helliker and Richter [1] to calculate leaf water oxygen isotope enrichment relative to source water (Δ_leaf_) under two assumptions: 1) the ε_bio_ is constant and equal to 27‰, as it was assumed in their study, and 2) ε*_bio_* varies with temperature according to our polynomial fit. We used equation 1 to calculate Δ_leaf_ by rearranging the terms of equation 1:
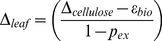
(4)For the calculations in which ε*_bio_* varied with temperature, we assumed both leaf and trunk had the same average temperature as the mean annual temperature. If the M.A.T. was below 5°C, we assumed values of *ε_bio_* to be that at 5°C. In the above calculation we exclude three points out of 70 in a site which in the Helliker and Richter [1] study were shown to be outliers. We compared the above values of Δ*_leaf_* with those actually measured in a global samples set from the Moisture Isotopes the Biosphere and Atmosphere (MIBA) network sponsored by the International Atomic Energy Agency [23]. The description and sampling months for each of the sites are shown in Table 1.

**Table 1:** Leaf and Stem water sampling stations of the MIBA network used to calculate isotopic enrichment of leaf water in Figure 3.

Location	M.A.T. (°C)	Months Sampled
Brazil (Brasilia)	21	June, August, November, December, February, March and April (2006–2007)
Canada (Eastern Peatland)	5.8	June–Sptember (2005)
Canada (New Brunswick)	2.1	June–Sptember (2005)
Canada (Saskatchewa)	0.4	June–Sptember (2005)
Canada (Western Peatland)	2.1	June–Sptember (2005)
Israel (Yatir)	22	Jan–Dec (2004–2005)
China (Daxing)	11.5	May–Nov (2005)
Russia (Siberia)	−5	June–September (2005)
Czechoslovakia (North Moravia)	5	May–September (2005)
Czechoslovakia (South Bohemia)	5	May–September (2005)
Ecuador (Yasuni National Forest)	25.5	Jan–December (2005–2007)
United Kingdom (Swansea)	12	February–April (2005)

Table gives the location of leaf and stem water sample collection from the M.I.B.A. network [23], mean annual temperature (M.A.T.) and months sampled. Note that the months sampled in colder habitats are usually considered the months with the greatest photosynthetic activity. Locations with multi-year sampling might have missing months during any one year, but all the months of the year were sampled at least once.

## Results

The δ^18^O values of cellulose from the seed germination experiment were highly correlated with those of the water having a slope (*p*_ex_) averaging 0.42±.01 and an average intercept of 31.03±0.21 (Table 2; see also Table S1). The slopes of the cultures at 5 and 10°C were slightly higher than those of higher temperature, but the differences were not significant (Table 2). After the Bonferroni correction, only slopes for cultures growing at 15 and 10°C were significantly different from each other. There were no significant differences between slopes from cultures growing at the extremes of temperature (5 and 30°C). A correlation analysis between slope and temperature did not show a significant trend in slope as a function of temperature (r = 0.73, P>0.05).

**Table 2:** Linear regression parameters between oxygen isotope ratios of seedling cellulose and culture water.

Temperature °C	5	10	15	20	25	30
**Linear Regression Intercept**	31.3	30.7	31.8	30.6	31.3	30.5
**Linear Regression Slope**	0.45±0.02	0.44±0.00	0.39±0.01	0.41±0.01	0.41±0.02	0.40±0.01
**r (correlation coefficient)**	1.00	1.00	1.00	1.00	1.00	1.00
**P**	<0.01	<0.01	<0.01	<0.01	<0.01	<0.01
**Differences in slope**	ab	a	b	ab	ab	ab

Parameters (slope and intercept) of linear regressions of the δ^18^O value of seedling cellulose on that of the culture water at various temperatures. Correlation coefficient and the significance are shown on the fourth and fifth row respectively. Difference of slope row (row 6) shows specific differences in the slopes. If they do not share a common letter, then slopes are significantly different.

**Figure 1:**
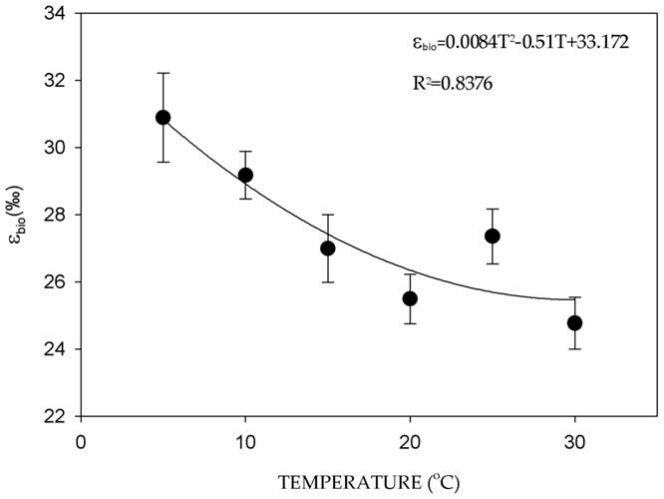
ε*_bio_* versus temperature. Average biochemical oxygen isotopic fractionation (ε_bio_±S.E.M., n = 5) in ‰ units for cellulose synthesized heterotrophically in wheat seedlings versus temperature (°C).

The average biochemical oxygen isotope fractionation for the seedling growth experiment varied from ∼25‰ at the highest temperature to ∼31‰ for the lowest temperature. The average ε*_bio_* varied less at temperatures between 20 and 30°C and increased more steeply at lower temperatures (Figure 1). There was a significant effect of temperature in the ε*_bio_* (F = 5.85, P = <0.01) but no effect of the culture water isotopic enrichment level (F = 0.7, P>0.05). A best fit polynomial regression of average ε*_bio_* on temperature gave a significant fit (r = 0.92, P<0.05).

**Figure 2:**
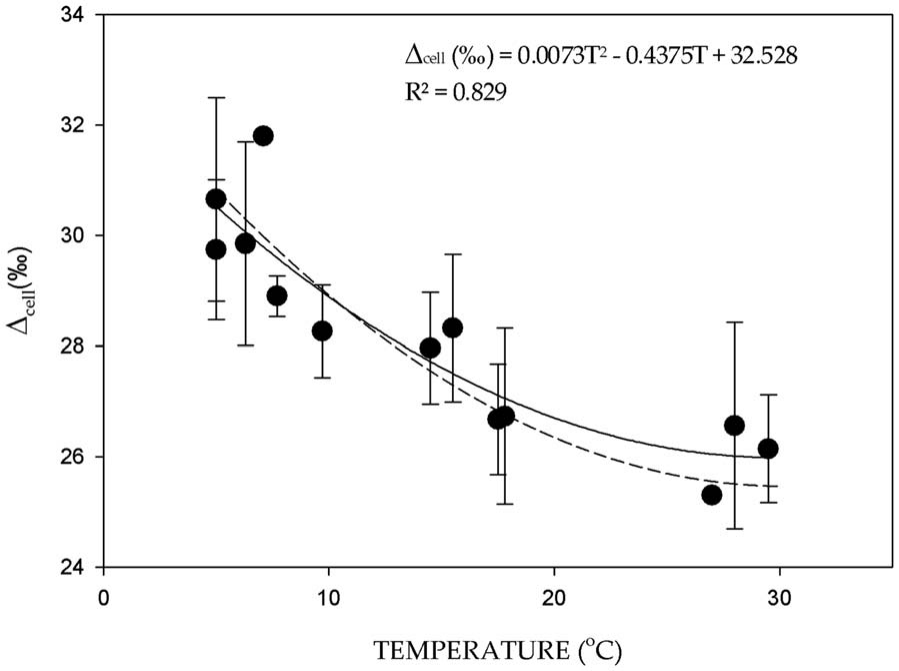
Aquatic Δ_cell_ versus temperature. Cellulose isotopic enrichment relative to source water (‰ units) for aquatic plants grown submerged from various studies [13], [18], [19]. Information for each data point is provided in the supplement (Table 2). Bold line is the best fit polynomial regression while the stippled line is for the best fit from wheat seedling experiment (Figure 1).

The biochemical oxygen isotope fractionation of aquatic and marine plants and organisms varied from 26 to 31‰ from lakes at lower latitudes having temperatures close to 30°C to lakes at cooler regions having temperature of 5°C respectively (Table S2). The fractionation was highly correlated with temperature yielding a polynomial regression equation similar to the one derived for the seedling growth experiment (Figure 2; also see Figure S1 for specific locations).

**Figure 3:**
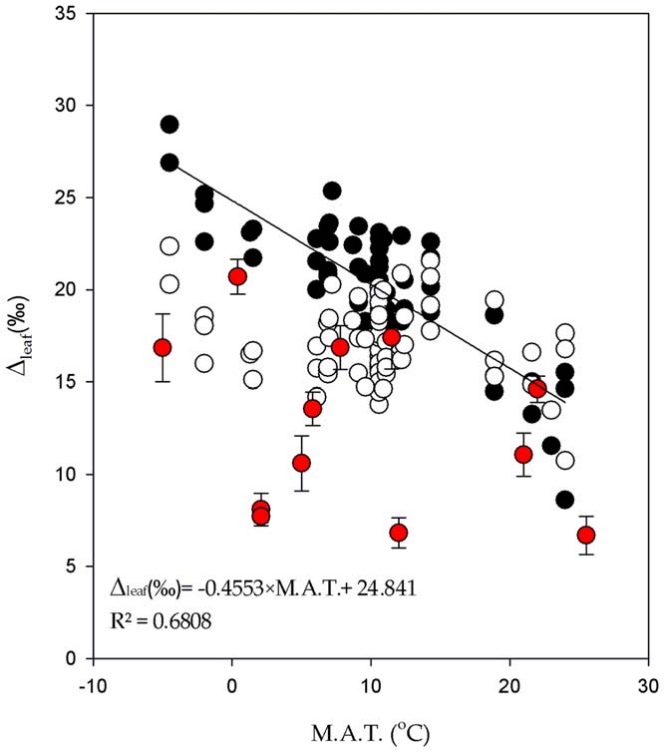
Δ_leaf_ versus mean annual temperature. Oxygen isotopic enrichment of leaf water relative to source water based on three types of calculations as described in the text: (•) based on a constant ε*_bio_* of 27‰; (○) based on a temperature sensitive ε*_bio_* adjusted to M.A.T.; (•) based on actual leaf water and stem water measurements. The relationship between Δ_leaf_ and M.A.T. was only significant with Δ_leaf_ calculated with a constant ε_bio_.

As expected and as proposed by Helliker & Richter [1] the Δ*_leaf_* increases with lower M.A.T. when one assumes a constant ε*_bio_* of 27‰ (Figure 3, black circles). On the other hand, Δ*_leaf_* calculated with a temperature sensitive ε*_bio_* indicated a constant Δ*_leaf_* averaging (17.1±0.3‰, empty circles) regardless of M.A.T. Observed Δ*_leaf_* from the MIBA samples indicated no specific trend as well (Figure 3, red circles; also see Table S3 and Figure S2 for specific location), with a greater degree of scatter and lower enrichment values averaging (12.2±1.3‰, red circles), but with many values matching the predictions of Δ*_leaf_* calculated with the temperature sensitive ε*_bio_*.

## Discussion

Our first hypothesis regarding differences in *p_ex_* with temperatures was shown false (Table 2). Significant temperature effects in *p_ex_* were only observed between 10 and 15°C, nor was there a significant trend in *p_ex_* as a function of temperature. One would expect that since *p_ex_* is a function of the futile cycle: glucose – 6 P <$>\raster(70%)="rg1"<$>fructose 1–6 P <$>\raster(70%)="rg1"<$>Gylceraldehyde-3 P +Dihydroxyacetone P; that it would occur to a greater extent at higher temperatures than lower temperatures. However, what actually determines the extent of exchange during this futile cycle would be the lifetime of each of these components, particularly the triose phosphates; i.e. the greater the lifetime, the greater the exchange. Although high temperatures could increase the reaction rates which lead to the above intermediates, it will also drive respiratory rates and possibly cellulose synthesis up, which consumes these intermediates and decreases the lifetime of the exchange intermediates and the oxygen isotope exchange. It is likely that any increase in the futile cycle rate is offset by a decrease in the lifetime of the futile cycle intermediates, thus causing *p_ex_* to remain constant with temperature.

In contrast to *p_ex_*, there was a significant increase in ε*_bio_* with a decrease in temperature (Figure 1). This would be the expected pattern for an equilibrium reaction. One of the principal reactions responsible for the exchange of oxygen between carbohydrates and water during cellulose synthesis is the carbonyl hydration reaction [3], [7]. Previous measurements for the isotopic fractionation during the carbonyl hydration reaction in a model molecule (acetone) indicates values similar to the fractionation between cellulose and water as well as a significant temperature effect at higher temperatures [7]. Equilibrium fractionation factors are often a direct function of the ratio of the partition coefficients for each isotopes which become closer to each other with an increase in temperature [12], hence the observed decrease in fractionation with higher temperature for equilibrium fractionation factors (see Clark & Fritz [24] for a compilation of equilibrium factors). Our results explain why a previous study using 6 different species of plants failed to observe a temperature effect [14]. Zhou [14] observed no temperature effect for ε*_bio_* for plants grown at 30 and 20°C. This range of temperature is precisely the range of temperature with the least effect on the biochemical fractionation (Figure 1).

Published observations of oxygen isotope enrichment of cellulose relative to ambient water of aquatic plants at different temperatures are consistent with our experimental results; there is an increase in enrichment with a decrease in temperature (Figure 2). In the case of aquatic plants, because they are submerged and there is no transpiration, the oxygen isotope enrichment of cellulose relative to the source water is the same as the biochemical fractionation (i.e. Δ*_leaf_* in equation 1 is equal to 0). The best fit relationship is also a second order polynomial fit having r = 0.91 and p<0.01 compared to the linear fit (r = 0.88and p<.01). The polynomial fit for the ε*_bio_* from the seed germination experiments fits remarkably well with the relationship observed for aquatic plants (Figure 2). Because the Calvin cycle end product (Glyceraldehyde-3 P) is one of the intermediates of the futile cycle mentioned above, it is likely that biochemical fractionation for heterotrophic and autotrophic cellulose synthesis are very similar, as it is often assumed in the literature [5]. Measurements of translocated sucrose which is autotrophically synthesized, indeed, show that the oxygen isotope ratios of autotrophically synthesized sucrose is ∼27‰ more enriched than that of the leaf lamina water and similar to heterotrophic cellulose synthesis [25].

Estimation of leaf water ^18^O enrichment based on a constant ε*_bio_* of 27‰ with the previously published data set predict that Δ*_leaf_* would increase with lower M.A.T. (r = 0.82, p<0.01). Helliker & Richter [1], therefore, invoked a greater vapor pressure gradient in colder regions, which can only be achieved by a greater increase in leaf temperature compared to ambient temperature during biomass production. However, when we calculate Δ*_leaf_* using the temperature sensitive ε*_bio_*, no trends of Δ*_leaf_* in relation to M.A.T. (r = 0.24, p>0.05) were observed. The Δ*_leaf_* extrapolated using the temperature sensitive ε*_bio_* is similar to those calculated with the constant ε*_bio_* of 27‰ at warmer temperatures but lower at cooler temperatures. The calculations of actual Δ*_leaf_* based on a global sample set initiated by the International Atomic Energy Agency (MIBA) gives an even greater discrepancy between observed Δ*_leaf_* as a function of M.A.T. and those based on a constant ε*_bio_*, although there were several values similar to those extrapolated based on a temperature sensitive ε*_bio_*. We have no experimental data to explain some of the lower than expected values of leaf water isotope enrichment for the MIBA samples, but speculate here that lower values were caused by the presences of non lamina leaf water in the MIBA samples. Previous studies showed a good correlation between the oxygen isotope ratios of phloem carbohydrates with lamina water. The authors of this study [25] went through considerable trouble to remove the main veins from leaves and only extract lamina water. Although the leaf sampling protocol for MIBA advises the removal of petiole and the main vein, it is likely that second order veins were not removed during leaf sampling leading to a more depleted leaf water values than the leaf lamina. In addition, because the MIBA samples involved spot sampling it would likely lead to greater variation in the isotopic composition than that extrapolated from tree trunk cellulose, which integrates leaf water over a considerable period of time and photosynthesis occurring throughout the whole plant.

Our data on the temperature sensitivity of the biochemical fractionation during cellulose synthesis indicates that there is an increase in ε*_bio_* with lower temperature. Hence, it is not necessary to invoke greater leaf water enrichment at colder habitat to explain the greater cellulose isotopic enrichment at high latitudes. An analysis of leaf and stem water samples from several latitudes, indeed, show no trending increase in leaf isotopic enrichment with a decrease in M.A.T. It is well recognized that photosynthesis in plants is limited to a temperature window. For example, plants in boreal climates do not have significant photosynthetic biomass production during the winter. Likewise, it is also well known that in hot climates plant photosynthetic responses include the so called “mid-day depression”, with stomatal closure and lower photosynthesis, which avoids the hotter and dryer times of the day [26]. However, there is also a large body of literature showing that plants native to a colder habitat will have a lower temperature photosynthetic optima [22]. Further, many plants can acclimate to different temperatures so as to have optimal photosynthesis over a wide range of temperatures [22]. Acclimation is an important and likely costly evolutionary process which would be wasted if plants produce the bulk of their biomass at a constant temperature of 21.4°C across a wide range of biomes.

## Supporting Information

Figure S1 **Aquatic Δ_cell_ versus temperature.** Cellulose oxygen isotope enrichment relative to source water (‰ units) for submerged aquatic plants from various studies [13], [18], [19]. Bold line is the best fit polynomial regression while the stippled line is for the best fit from wheat seedling experiment. Data points are indexed according to the graph code on Table S2.
(TIF)

Figure S2 **Δ_leaf_ versus mean annual temperature.** Oxygen isotopic enrichment of leaf water relative to source water based on three types of calculations as described in the text: (•) based on a constant ε_bio_ of 27‰; (○) based on a temperature sensitive ε_bio_ adjusted to M.A.T.; (•) based on actual leaf water and stem water measurements. The relationship between Δ_leaf_ and M.A.T. was only significant with Δ_leaf_ calculated with a constant ε_bio_. Data points based on actual measurements are indexed in Table S3.
(TIF)

Table S1 **Experimental raw data for wheat seedling experiment.** Data in columns are Temp(°C) = temperature, (δ^18^O_water_) = Culture water oxygen isotope ratios, two replicates were taken (rep-1 and rep-2) and the mean determined from these two replicates, (δ^18^O_cell_) = oxygen isotope ratios of seedling cellulose for each culture, the slope and intercept for the relationship between δ^18^O_cell_ and the δ^18^O_water_ , Ind. ε_bio_ = biochemical oxygen isotope ratio fractionation of cellulose relative to the oxygen isotope ratio of the culture water, calculated as in equation 3 in text, Mean (ε_bio_) = average of values in the previous column, SEM = Standard error of the mean. Table also shows the ε_bio_ and SEM calculated by the slope and intercept method as in reference 3.
(PDF)

Table S2 **Oxygen isotope ratios of aquatic plants and their ambient water from various locations.** Data in columns indicates the location of collection, average growing temperature, species, δ^18^O value of the water and of the cellulose respectively, the oxygen isotope enrichment of cellulose relative to the source water for each species in a location, the average cellulose oxygen isotope enrichment for each location and the respective standard deviation and the data source.
(PDF)

Table S3 **Summary of leaf water isotopic enrichment relative to stem water.** Mean annual temperature and mean leaf water oxygen isotope ratio enrichment relative to that of stem water and the respective standard error of the mean at various locations in the Moisture Isotope in the Biosphere network (International Atomic Energy Agency, http://www-naweb.iaea.org/napc/ih/IHS_resources_miba.html). Data is coded (Graph Code) to be referred by Figure S2.
(PDF)
