# Supplementary material for: Divergent Biochemical Fractionation, Not Convergent Temperature, Explains Cellulose Oxygen Isotope Enrichment across Latitudes
Source: PLoS One. 2011 Nov 21;6(11):e28040. doi: 10.1371/journal.pone.0028040 (PMC3221677; doi:10.1371/journal.pone.0028040)
Supplement: Table S1 — Experimental raw data for wheat seedling experiment. Data in columns are Temp(°C) = temperature, (δ18Owater) = Culture water oxygen isotope ratios, two replicates were taken (rep-1 and rep-2) and the mean determined from these two replicates, (δ18Ocell) = oxygen isotope ratios of seedling cellulose for each culture, the slope and intercept for the relationship between δ18Ocell and the δ18Owater , Ind. εbio = biochemical oxygen isotope ratio fractionation of cellulose relative to the oxygen isotope ratio of the culture water, calculated as in equation 3 in text, Mean (εbio) = average of values in the previous column, SEM = Standard error of the mean. Table also shows the εbio and SEM calculated by the slope and intercept method as in reference 3. (PDF) [file pone.0028040.s003.pdf]

Table S1. Experimental raw data for wheat seedling experiment. Data in columns are Temp(°C) = temperature, ($\delta^{18}O_{water}$)=Culture water oxygen isotope ratios, two replicates were taken (rep-1 and rep-2) and the mean determined from these two replicates, ($\delta^{18}O_{cell}$)= oxygen isotope ratios of seedling cellulose for each culture, the slope and intercept for the relationship between $\delta^{18}O_{cell}$ and the $\delta^{18}O_{water}$, Ind. ε_{bio} = biochemical oxygen isotope ratio fractionation of cellulose relative to the oxygen isotope ratio of the culture water, calculated as in equation 3 in text, Mean (ε_{bio})= average of values in the previous column, SEM= Standard error of the mean. Table also shows the ε_{bio} and SEM calculated by the slope and intercept method as in reference 3.

	($\delta^{18}O_{water}$)							Average of Individual		Calculated by Intercept	
Temp. (°C)	rep -1	rep-2	Mean	($\delta^{18}O_{cell}$)	Slope	Intercept	Ind. ε_{bio}	Mean(ε_{bio})	SEM	ε_{bio}	SEM
5	-0.8	0.0	-0.4	31.9			30.1				
5	18.4	18.4	18.4	39.4			29.3				
5	36.6	36.2	36.4	46.1			27.2				
5	54.9	54.9	54.9	56.5			33.5				
5	71.2	71.8	71.5	63.8	0.45	31.30	34.3	30.9	1.3	28.7	0.866
10	0.3	0.2	0.3	30.9			27.3				
10	18.1	18.2	18.2	38.8			28.1				
10	36.5	36.5	36.5	47.0			29.3				
10	54.3	54.9	54.6	54.8			29.8				
10	72.0	71.8	71.9	62.8	0.44	30.79	31.4	29.2	0.7	27.5	0.942
15	-0.7	-0.8	-0.8	31.8			30.4				
15	14.9	14.9	14.9	37.1			27.3				
15	35.9	35.9	35.9	45.9			27.3				
15	54.3	54.4	54.4	53.0			25.7				
15	70.4	70.5	70.5	59.2	0.39	31.79	24.4	27.0	1.0	29.3	0.277
20	-0.9	-0.6	-0.8	30.8			27.9				
20	14.2	14.6	14.4	36.2			25.7				
20	35.0	34.9	35.0	44.2			24.3				
20	53.5	53.9	53.7	51.9			23.7				

20	69.6	70.2	69.9	59.6	0.41	30.57	25.9	25.5	0.7	26.4	0.5035
25	-0.6	-0.3	-0.5	31.1			28.4				
25	16.6	16.2	16.4	37.1			25.9				
25	33.9	34.1	34.0	46.1			29.5				
25	47.3	50.8	49.1	51.7			27.9				
25	68.5	67.7	68.1	58.5	0.41	31.25	25.1	27.4	0.8	28.1	0.649
30	0.7	0.6	0.7	31.2			27.6				
30	17.8	17.9	17.9	37.5			25.3				
30	36.9	37.0	36.9	44.6			23.2				
30	53.0	52.8	52.9	51.6			23.9				
30	72.9	72.9	72.9	60.0	0.40	30.50	23.9	24.8	0.8	26.1	0.4255
