# Supplementary material for: Divergent Biochemical Fractionation, Not Convergent Temperature, Explains Cellulose Oxygen Isotope Enrichment across Latitudes
Source: PLoS One. 2011 Nov 21;6(11):e28040. doi: 10.1371/journal.pone.0028040 (PMC3221677; doi:10.1371/journal.pone.0028040)
Supplement: Table S2 — Oxygen isotope ratios of aquatic plants and their ambient water from various locations. Data in columns indicates the location of collection, average growing temperature, species, δ18O value of the water and of the cellulose respectively, the oxygen isotope enrichment of cellulose relative to the source water for each species in a location, the average cellulose oxygen isotope enrichment for each location and the respective standard deviation and the data source. (PDF) [file pone.0028040.s004.pdf]

Table S2. Oxygen isotope ratios of aquatic plants and their ambient water from various locations. Data in columns indicates the location of collection, average growing temperature, species, $\delta^{18}O$ value of the water and of the cellulose respectively, the oxygen isotope enrichment of cellulose relative to the source water for each species in a location, the average cellulose oxygen isotope enrichment for each location and the respective standard deviation and the data source.

Location	Temp. (°C)	Species	($\delta^{18}O_{water}$)	($\delta^{18}O_{cell}$)	(Δ_{cell})	Mean (Δ_{cell})	σ	Graph Code	Ref #
Villeperdu, France	17.8	*Myriophyllum sp.*	-1.2	24.2	25.4				18
Lespuau, France	17.8	*Alisma plantago*	-3.5	25	28.5				18
Lespuau, France	17.8	*Potamogeton crispus*	-3.5	22.8	26.3	26.7	1.6	1	18
Imbabura, Ecuador	9.7	*Myriophyllum sp.*	-7.4	21.3	28.7				18
Imbabura, Ecuador	9.7	*Eleocharis acicularis*	-7.4	19.9	27.3				18
Imbabura, Ecuador	9.7	*Eleocharis acicularis*	-7.4	21.4	28.8	28.3	0.8	2	18
Cotapaxi, Ecuador	7.7	*Elatina sp.*	-8.9	20.4	29.3				18
Cotapaxi, Ecuador	7.7	*Potamogeton sp.*	-8.9	19.8	28.7				18
Cotapaxi, Ecuador	7.7	*Eleocharis maculosa*	-8.9	19.6	28.5				18
Cotapaxi, Ecuador	7.7	*Equisetum boyotensis*	-8.9	20.2	29.1	28.9	0.4	3	18
Junin, Peru	6.3	*Myriophyllum quitensis*	-9.5	17.7	27.2				18
Junin, Peru	6.3	*Potamogeton panamensis*	-11.0	20.4	31.4				18
Lago Calzado, Peru	6.3	*Calitriche nubigena*	-16.4	14.3	30.7				18
Lago Calzado, Peru	6.3	*Isoetes Palmerii*	-16.4	13.7	30.1	29.9	1.8	4	18
lago Chisaca, Colombia	7.1	*Crassula palludosa*	-10.6	21.2	31.8	31.8		5	18
Lago Gatun, Panama	27	*Hydrilla verticillata*	-3.9	21.4	25.3	25.3		6	18
Miami, Florida, USA	28	*Utricularia sp.*	2.4	26.2	23.8				18
Miami, Florida, USA	28	*Eleocharis acicularis*	-1.6	27.2	28.8				18
Miami, Florida, USA	28	*Cabomba caroliniana*	-1.6	24.3	25.9				18
Miami, Florida, USA	28	*Hydrilla verticillata*	-1.6	25.8	27.4				18

Miami, Florida, USA	28	*Najas guadalupensis*	-1.6	25.3	26.9	26.6	1.9	7	18
Aquarium	5	*Plagiothecium denticulatum*	-22.2	8.6	30.8				19
Aquarium	5	*Plagiothecium denticulatum*	-18.9	11.9	30.8				19
Aquarium	5	*Plagiothecium denticulatum*	-16.1	14	30.1				19
Aquarium	5	*Plagiothecium denticulatum*	-6.3	22.9	29.2				19
Aquarium	5	*Plagiothecium denticulatum*	4.1	31.9	27.8	29.7	1.3	8	19
B9413 (lake ID)	5	*Moss not identified*	-16.3	14.6	30.85				19
B9437	5	*Moss not identified*	-16.8	12.1	28.91				19
B9437	5	*Moss not identified*	-16.8	13.3	30.11				19
B9437	5	*Moss not identified*	-16.8	13.9	30.71				19
B9441	5	*Moss not identified*	-18.1	9.8	27.89				19
B9441	5	*Moss not identified*	-18.1	11.1	29.19				19
B9441	5	*Moss not identified*	-18.1	12.1	30.19				19
B9441	5	*Moss not identified*	-18.1	13.2	31.29				19
B9442	5	*Moss not identified*	-17.5	9.2	26.67				19
B9442	5	*Moss not identified*	-17.5	12.9	30.37				19
B9448	5	*Moss not identified*	-21.1	7.2	28.28				19
B9448	5	*Moss not identified*	-16.3	15.1	31.35				19
B9448	5	*Moss not identified*	-14.2	15.3	29.5				19
B9448	5	*Moss not identified*	-14.2	15.5	29.7				19
B9448	5	*Moss not identified*	-14.8	16.3	31.1				19
B9452	5	*Moss not identified*	-14.8	15.8	30.6				19
B9464	5	*Moss not identified*	-15.1	16.1	31.17				19
B9472	5	*Moss not identified*	-18.7	10.2	28.91				19
B9512	5	*Moss not identified*	-19.2	9.1	28.3				19
B9520	5	*Moss not identified*	-17.0	13.9	30.93				19
B9539	5	*Moss not identified*	-20.1	9.7	29.76				19
B9544	5	*Moss not identified*	-23.5	7.2	30.74				19
B9545	5	*Moss not identified*	-20.4	14.9	35.32				19
B9548	5	*Moss not identified*	-16.8	10	26.81				19

Brevoort Water L	5	*Moss not identified*	-14.7	15.7	30.4				19
Brevoort Water L	5	*Moss not identified*	-14.7	16	30.7				19
Brevoort Water L	5	*Moss not identified*	-14.7	16.8	31.5				19
Brevoort Water L	5	*Moss not identified*	-14.7	16.9	31.6				19
Brevoort Water L	5	*Moss not identified*	-14.7	17	31.7				19
Brevoort Water L	5	*Moss not identified*	-14.7	18.2	32.9				19
Brevoort Water L	5	*Moss not identified*	-14.7	18.7	33.4				19
Camp Lake	5	*Moss not identified*	-16.0	15.9	31.86				19
Dyer Lower	5	*Moss not identified*	-16.4	16.6	33				19
Dyer Lower	5	*Moss not identified*	-16.4	16.9	33.3				19
Hall Beach	5	*Moss not identified*	-16.5	16	32.5				19
Robinson	5	*Moss not identified*	-15.7	16.3	31.99	30.7	1.8	9	19
British Colombia, Canada	14.5	*Macrocystis sp.*	-1.7	26.3	28				13
British Colombia, Canada	14.5	*Halocynthia igaboja*	-1.7	27	28.7				13
British Colombia, Canada	14.5	*Styela montereyensis*	-1.7	28.4	30.1				13
British Colombia, Canada	14.5	*Laminaria sp.*	-1.7	25.5	27.2				13
British Colombia, Canada	14.5	*Ascidia paratropa*	-1.7	26	27.7				13
British Colombia, Canada	14.5	*Boltenia villosa*	-1.7	25.8	27.5				13
British Colombia, Canada	14.5	*Cnemidocarpa finmarkiensis*	-1.7	25.8	27.5				13
British Colombia, Canada	14.5	*Halocynthia aurantium*	-1.7	25.3	27	28.0	1.0	10	13
Puerto Rico, USA	27.5	*Aplidium lobatum*	0.2	25.6	25.4				13
Puerto Rico, USA	27.5	*Ecteinascidia turbinata*	0.2	25.1	24.9				13
Florida, USA	29.5	*Thalassia testudinum*	0.4	28	27.6				13
Florida, USA	29.5	*Amaroucium stellatum*	0.4	26.1	25.7				13
Florida, USA	29.5	*Didemum candidum*	0.4	26.2	25.8				13
Florida, USA	29.5	*Molgula occidentalis*	0.4	27.6	27.2				13
Florida, USA	29.5	*Styela plicata*	0.4	26.8	26.4	26.1	1.0	11	13
California, USA	15.5	*Gelidium robustum*	-1.6	27.9	29.5				13
California, USA	15.5	*Macrocystis sp.*	-1.6	25.8	27.4				13
California, USA	15.5	*Ulva sp.*	-1.6	24.2	25.8				13

California, USA	15.5	*Phyllospadix torreyi*	-1.6	27.8	29.4				13
California, USA	15.5	*Zostera marina*	-1.6	27.3	28.9				13
California, USA	15.5	*Ciona intestinalis*	-1.6	26.8	28.4				13
California, USA	15.5	*Metandrocarpa dura*	-1.6	25.4	27				13
California, USA	15.5	*Pyura haustor*	-1.6	28.2	29.8				13
California, USA	15.5	*Styela montereyensis*	-1.6	27.1	28.7	28.3	1.3	12	13
Massachusetts, USA	17.5	*Ascophyllum nodosum*	-1.2	25.1	26.3				13
Massachusetts, USA	17.5	*Chondria tenuissima*	-1.2	24.2	25.4				13
Massachusetts, USA	17.5	*Chondrus crispus*	-1.2	25.6	26.8				13
Massachusetts, USA	17.5	*Fucus vesiculosus*	-1.2	25.8	27				13
Massachusetts, USA	17.5	*Laminaria agardhii*	-1.2	25.5	26.7				13
Massachusetts, USA	17.5	*Polysiphonia nigrescens*	-1.2	25.6	26.8				13
Massachusetts, USA	17.5	*Ulva lactuca*	-1.2	24.2	25.4				13
Massachusetts, USA	17.5	*Thalassia sp.*	-1.2	26.8	28				13
Massachusetts, USA	17.5	*Amaroucium stellatum*	-1.2	26.4	27.6				13
Massachusetts, USA	17.5	*Ciona intestinalis*	-1.2	26.5	27.7				13
Massachusetts, USA	17.5	*Didemnum albidum*	-1.2	23.7	24.9				13
Massachusetts, USA	17.5	*Molgala sp.*	-1.2	26.2	27.4				13
Massachusetts, USA	17.5	*Styela Partita*	-1.2	26.5	27.7	26.7	1.0	13	13
