# Supplementary material for: Divergent Biochemical Fractionation, Not Convergent Temperature, Explains Cellulose Oxygen Isotope Enrichment across Latitudes
Source: PLoS One. 2011 Nov 21;6(11):e28040. doi: 10.1371/journal.pone.0028040 (PMC3221677; doi:10.1371/journal.pone.0028040)
Supplement: Table S3 — Summary of leaf water isotopic enrichment relative to stem water. Mean annual temperature and mean leaf water oxygen isotope ratio enrichment relative to that of stem water and the respective standard error of the mean at various locations in the Moisture Isotope in the Biosphere network (International Atomic Energy Agency, http://www-naweb.iaea.org/napc/ih/IHS_resources_miba.html). Data is coded (Graph Code) to be referred by Figure S2. (PDF) [file pone.0028040.s005.pdf]

Table S3. Summary of leaf water isotopic enrichment relative to stem water. Mean annual temperature and mean leaf water oxygen isotope ratio enrichment relative to that of stem water and the respective standard error of the mean at various locations in the Moisture Isotope in the Biosphere network (International Atomic Energy Agency, ://www-naweb.iaea.org/napc/ih/IHS_resources_miba.html). Data is coded (Graph Code) to be referred by the next figure.

Location	MAT	Mean Δ_{leaf}	SEM	Graph Code
Brasilia-Brazil	21.0	11.1	1.2	1
EP-Canada (Eastern Peatland)	5.8	13.6	0.9	2
NB-Canada-(New Brunswick site)	2.1	8.1	0.9	3
SK-Canada-(Saskatchewan site)	0.4	20.7	0.9	4
WP-Canada (Western Peatland)	2.1	7.7	0.3	5
Yatir -Israel	22.0	14.6	0.7	6
Daxing -China	11.5	17.4	1.7	7
Siberia -Russia	-5.0	16.9	1.8	8
Bily Kriz-Czech Republic	5.0	10.6	1.5	9
Ceske Budejovice-Czech Republic	7.8	16.9	1.2	10
Yasuni National Forest-Ecuador	25.5	6.7	1.0	11
Swansea-United Kingdom	12.0	6.8	0.8	12
